# Repeatability and comparison of new Corvis ST parameters in normal and keratoconus eyes

**DOI:** 10.1038/s41598-019-51502-4

**Published:** 2019-10-25

**Authors:** Kaili Yang, Liyan Xu, Qi Fan, Dongqing Zhao*, Shengwei Ren*

**Affiliations:** Henan Provincial People’s Hospital, Henan Eye Hospital, Henan Eye Institute, People’s Hospital of Zhengzhou University, School of Clinical Medicine, Henan University, Zhengzhou, 450003 China

**Keywords:** Eyelid diseases, Diagnostic markers

## Abstract

To evaluate the repeatability of corneal biomechanical parameters in normal and keratoconus eyes, and explore factors that affects the repeatability, and further assess the diagnostic ability of new parameters. Seventy-seven keratoconus eyes of 47 patients and 77 right eyes of 77 normal subjects were recruited in current study. All participants received three repeated measurements with 2 to 5 minutes interval. The interclass correlation coefficient (ICC), Cronbach’ α and repeatability coefficient (RC) were evaluated. The liner regression analysis was used to identify factors that affect the repeatability, and linear mixed effects model was performed to compare the parameters differences. The receiver operating characteristic (ROC) curve was used to evaluate the diagnostic ability of new parameters. Eighteen parameters in normal eyes and twenty-two parameters in keratoconus eyes showed excellent repeatability (ICC ≥ 0.90). Age, axial measurement (AL), spherical equivalent, astigmatism, gender, mean keratometry (Kmean), intraocular pressure (IOP) and central corneal thickness (CCT) could affect the repeatability of new Corvis ST parameters. Compared with normal eyes, the Ambrósio’s Relational Thickness horizontal (ARTh), biomechanical corrected IOP (bIOP), stiffness parameter at first applanation (SP A1) were low and the Max Inverse Radius, deformation amplitude (DA) Ratio Max [2 mm], Pachy Slope, DA Ratio Max [1 mm], Integrated Radius and Corvis Biomechanical Index (CBI) were high in keratoconus eyes (All *P* < 0.05). Both ARTh and CBI had high Youden index (0.870), and the corresponding cut-off values were 379.29 and 0.44. The repeatability of Corvis ST parameters was acceptable both in normal and keratoconus eyes, and new parameters could effectively diagnose keratoconus eyes from normal eyes.

## Introduction

Corneal biomechanics, which reflects the corneal deformation in response to external forces, is a determinant of corneal shape^1^. Recent studies have demonstrated that corneal biomechanics played an important role in assessing glaucoma^2^ and detecting patients with corneal ectasia diseases especially for keratoconus^3^. Keratoconus is a progressive corneal disorder that could modify the normal architecture into a conical shape and lead to corneal apex thinning^4^. As a non-inflammatory disease, Keratoconus usually happens in adolescence and progresses until the third or fourth decade of life, and results in serious vision loss in advanced stages^5^. Recent study reported the corneal tomographic or topographic maps could help to diagnose keratoconus in the alteration of corneal shape^6^. However, the diagnoses for early and subclinical keratoconus presenting few or no symptom are still difficult^7^. Previous study found that the abnormal biomechanics occurred before the keratoconus patients had tomographic changes and clinical symptoms, and the corneal biomechanical parameters could be detected by ophthalmology device^8^. In addition, the corneal refractive surgery is widely accepted all over the world, and the detection of abnormal biomechanics before refractive surgery is particularly important to avoid complications^9^. Therefore, it is crucial to estimate the characters of corneal biomechanical parameters.

The Corneal Visualisation Scheimpflug Technology (Corvis-ST) is a relatively new noncontact tonometer, making it available to estimate the corneal biomechanical properties^10,11^. Previous studies reported that the repeatability of corneal biomechanical parameters was acceptable in normal eyes^12–15^. While limited study found the repeatability of intraocular pressure (IOP), central corneal thickness (CCT) and deformation amplitude were adequate in normal and keratoconus eyes^16^. In addition, the new parameters that improved smoothing algorithm and edge detection have been used in clinical application with the improvement of software. The new Corvis ST Software provides parameters such as the maximum value of the ratio between deformation amplitude at the apex 1 mm and 2 mm from central cornea (DA Ratio Max [1 mm], DA Ratio Max [2 mm]), biomechanical corrected IOP (bIOP), Ambrósio’s Relational Thickness horizontal (ARTh), stiffness parameter at first applanation (SP A1), Corvis Biomechanical Index (CBI)^17,18^. Recent study found that these new parameters had detectability for distinguishing keratoconus eyes from normal eyes^18^. However, it had not been reported that the repeatability of these new parameters in keratoconus. Even though repeatability of older parameters has been well established using the Corvis ST, it cannot be assumed that the newer parameters given by the manufacturer’s software are also repeatable. Therefore, the current study aimed to assess the repeatability of the Corvis ST parameters, and explore the factors that influenced the repeatability of new parameters. In addition, we also investigated the differences of parameters between normal and keratoconus eyes, and further evaluated the diagnostic ability of new parameters in detecting keratoconus from normal eyes.

## Methods

### Study subjects

A prospective, randomized comparative study was performed. Normal subjects and keratoconus patients at Henan Eye Hospital & Henan Eye Institute between September 2017 and December 2018 were enrolled. The diagnosis of keratoconus was based on the following criteria: asymmetric bowtie pattern with or without skewed axes revealed by corneal topography or keratoconus sign detected by slit-lamp examination, such as localized stromal thinning, conical protrusion, Vogt’s striae, Fleischer’s ring or anterior stromal scar^19^. Keratoconus severity was classified according to the Keratoconus Severity Score Ranking Scheme^20^. The mild or moderate keratoconus patients without anterior stromal scar and previous ocular surgery, such as corneal collagen cross-linking, intracorneal ring segments or corneal transplantation, were recruited in current study. Subjects eligible for refractive surgery with spherical equivalent <8.00 diopters (D), corneal astigmatism <1.50 D, the value of Best Corrected Visual Acuity (BCVA) in LogMAR ≤ 0.1 were enrolled as the control group. The exclusion criteria was that eyes with pre-existing corneal pathology or previous ocular surgery, rigid contact lens wears within 4 weeks, soft contact lens wears within 2 weeks, serious diabetes, and other eye diseases. Finally, 77 normal subjects (77 right eyes) and 47 mild or moderate keratoconus patients (77 keratoconus eyes) were recruited in current analysis.

### Examinations

The data of age, gender, and history of eye diseases was collected from medical records. All the measurements were obtained by the same experienced operator between 9:00 and 17:00. The ocular examinations such as visual acuity, ocular position, slit lamp, ophthalmoscope examination, mydriatic examination and lens insertion optometry were performed by a professional ophthalmologist. The axial measurement (AL) was measured using laser interferometer (IOLMaster, Carl Zeiss Meditec, Germany). The values of steep keratometry (Ks), flat keratometry (Kf), and mean keratometry (Kmean) were detected using Visante Omni anterior segment OCT (Carl Zeiss Jena GmBH, Germany).

**Figure 1:**
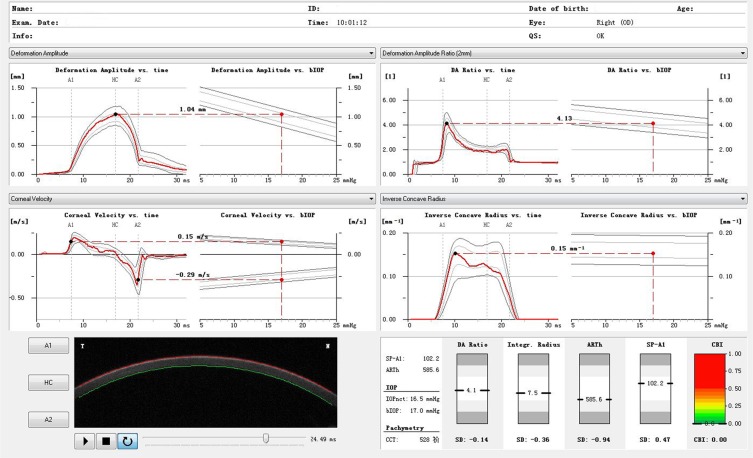
The Corvis ST parameters in normal eyes.

**Figure 2:**
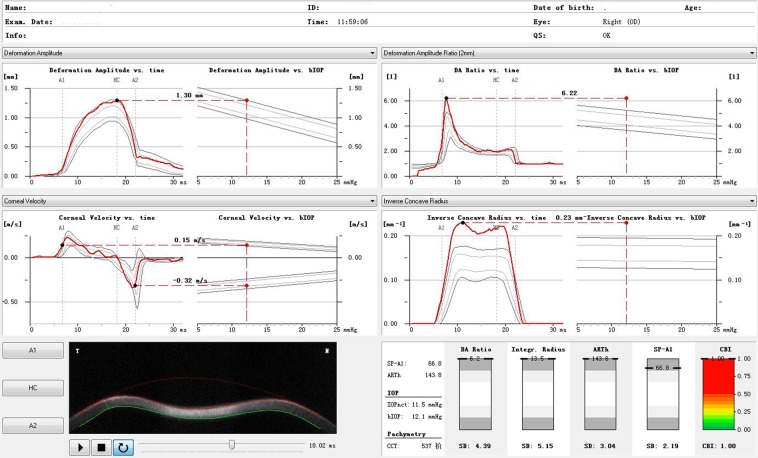
The Corvis ST parameters in keratoconus eyes.

All participants received three repeated Corvis ST (Oculus 72100, Wetzlar, Germany) measurements with 2 to 5 minutes interval. The instrument takes Scheimpflug images of the anterior segment at a rate of 4330 frames/s and collects approximately 140 horizontal section images. The following parameters were detected by Corvis ST: IOP, CCT, time from the initiation of air puff until the first applanation (A1T), second applanation (A2T) and maximum deformation (HCT); corneal velocity at the first (A1V) and sccond applanation (A2V); deformation amplitude at the first applanation (A1DA), second applanation (A2DA) and maximum deformation (HCDA); deflection length at the first applanation (A1DLL), second applanation (A2DLL) and maximum deformation (HCDLL); deflection amplitude at the first applanation (A1DLA), second applanation (A2DLA) and maximum deformation (HCDLA); deflection area at the first applanation (A1DLAr), second applanation (A2DLAr) and maximum deformation (HCDLAr); delta arc length at the first applanation (A1dArcL), second applanation (A2dArcL) and maximum deformation (HCdArcL); deformation amplitude Max (DA Max); peak distance (PD) and radius of curvature (Radius); max time and length at deflection amplitude (DLAMT, DLAML); max time and amplitude of whole eye movement (WEMT, WEMA); delta arc length max (dArcLM) and Pachy Slope. The current study used a beta version of Corvis ST Software. Except for the parameters enumerated above, the following new parameters were analyzed: DA Ratio Max [1 mm], DA Ratio Max [2 mm], Max Inverse Radius, bIOP, Integrated Radius, ARTh, SP A1, and CBI. The new Corvis ST parameters in normal eyes and keratoconus eyes were shown in Figs 1 and 2, respectively. A list of abbreviations has been shown in Supplementary Table 1.

### Statistical analysis

Continuous variables were described as mean ± standard deviation (SD), and compared by t test. To evaluate the repeatability, the interclass correlation coefficient (ICC) and 95% Confidence interval (*CI*), Cronbach’ α, and repeatability coefficient (RC) were calculated for each Corvis ST parameter^21^. The RC, adopted by the British Standards Institution as an indicator of measurement reliability, is based on the common within-subject SD (S _w_)^22^. In addition, coefficient of variation (CV) was calculated. To identify factors associated with the reliability of the measurements, we used subject SD (the SD of repeated measurements within each subject) as an indicator of measurement variability^23^. The liner regression analysis was used to explore the relationship between the subject SD and baseline factors such as age, AL, spherical equivalent, astigmatism, gender, Kmean, IOP and CCT. Linear mixed effects model was used to explore the differences of the Corvis ST parameters between two groups after adjusting for age, CCT and IOP. In addition, the receiver operating characteristic (ROC) curve was used to evaluate the diagnostic ability of Corvis ST parameters for distinguishing keratoconus eyes from normal eyes, and the values of cut-off, sensitivity, specificity, Youden index (the sum of sensitivity and specificity minus 1), area under the ROC curve (AUC) were recorded. The statistical analysis of the survey data was performed using SPSS 23.0 software package and MedCalc software, and *P* < 0.05 (two-tailed) was considered as statistically significant.

### Ethics approval and informed consent

This study has been registered at Chinese Clinical Trial Register (Registration number: ChiCTR-1900023046) and the design was approved by the Institutional Review Board of Henan Eye Hospital [ethical approval number: HNEECKY-2019 (5)]. Written informed consent was obtained from all patients.

## Results

### Demographics

Seventy-seven eyes of 77 normal subjects and 77 eyes of 47 keratoconus patients were recruited in the current study. The demographic data of participants was shown in Table 1. The mean age was 25.99 ± 3.71 years and 24.58 ± 5.19 years for normal eyes and keratoconus eyes, respectively, with no significant difference between them (*P* = 0.056). The absolute values of spherical equivalent, astigmatism, Ks, Kf and Kmean in keratoconus eyes were significantly higher than those in normal eyes (all *P* < 0.05).

**Table 1:** Demographic data of participants.

Parameters	Normal eyes	Keratoconus eyes	t	*P*
Age (years, mean ± SD)	25.99 ± 3.71	24.58 ± 5.19	1.930	0.056
Spherical equivalent (D, mean ± SD)	−4.89 ± 1.92	−7.65 ± 4.76	4.717	<0.001
Astigmatism (D, mean ± SD)	−0.53 ± 0.48	−2.85 ± 2.74	7.311	<0.001
AL (mm, mean ± SD)	25.33 ± 1.69	25.67 ± 1.89	−0.631	0.529
Ks (D, mean ± SD)	43.68 ± 1.37	49.37 ± 5.36	−9.020	<0.001
Kf (D, mean ± SD)	42.73 ± 1.23	46.69 ± 4.46	−7.509	<0.001
Kmean (D, mean ± SD)	43.21 ± 1.27	47.65 ± 6.13	−6.220	<0.001
BCVA (LogMAR)	0.004 ± 0.02	0.31 ± 0.34	−7.964	<0.001

AL: axial measurement, Ks: steep keratometry, Kf: flat keratometry, Kmean: mean keratometry, BCVA: Best Corrected Visual Acuity.

### Repeatability of Corvis ST parameters

Table 2 and 3 summarized the repeatability values of the Corvis ST parameters. In normal eyes, 18 of 39 parameters (46.15%) showed excellent repeatability (ICC ≥ 0.90), 10 parameters (25.64%) showed good (ICC ≥ 0.75), and 11 parameters (28.21%) showed poor to moderate repeatability (ICC < 0.75). Similarly, 22 parameters (56.41%) demonstrated excellent repeatability, 7 parameters (17.95%) showed good repeatability, and 10 parameters (25.64%) showed poor to moderate repeatability in keratoconus eyes.

**Table 2:** Repeatability of the Corvis ST parameters in normal eyes.

Parameters	CV (%)	Cronbach’ α	ICC(95% *CI*)	S _w_	RC
Established Variables					
IOP (mmHg)	0.11	0.93	0.93(0.90, 0.95)	0.73	2.02
CCT (µm)	0.06	0.99	0.99 (0.99, 0.99)	4.88	13.52
DA Max (mm)	0.08	0.91	0.91 (0.87, 0.94)	0.05	0.14
A1T (ms)	0.03	0.93	0.93 (0.90, 0.95)	0.09	0.25
A1V (m/s)	0.07	0.87	0.87 (0.81, 0.91)	0.01	0.03
A2T (ms)	0.01	0.92	0.92 (0.88, 0.94)	0.17	0.47
A2V (m/s)	−0.10	0.84	0.83 (0.76, 0.89)	0.02	0.06
HCT (ms)	0.02	0.67	0.67 (0.52, 0.78)	0.41	1.14
PD (mm)	0.05	0.92	0.92 (0.88, 0.95)	0.12	0.33
Radius (mm)	0.09	0.81	0.81 (0.72, 0.87)	0.44	1.22
A1DA (mm)	0.08	0.49	0.48 (0.24, 0.65)	0.01	0.03
HCDA (mm)	0.08	0.91	0.91 (0.87, 0.94)	0.05	0.14
A2DA (mm)	0.18	0.85	0.85 (0.78, 0.9)	0.04	0.11
A1DLL (mm)	0.10	0.72	0.73 (0.60, 0.82)	0.21	0.58
HCDLL (mm)	0.10	0.66	0.66 (0.50, 0.78)	0.61	1.69
A2DLL (mm)	0.28	0.62	0.62 (0.44, 0.75)	1.03	2.85
A1DLA (mm)	0.11	0.80	0.80 (0.71, 0.87)	0.00	0.00
HCDLA (mm)	0.10	0.92	0.92 (0.89, 0.95)	0.04	0.11
A2DLA(mm)	0.09	0.63	0.63 (0.46, 0.75)	0.01	0.03
DLAML (mm)	0.09	0.94	0.94 (0.91, 0.96)	0.04	0.11
DLAMT (ms)	0.02	0.20	0.21 (−0.16, 0.47)	0.64	1.77
WEMA (mm)	0.20	0.83	0.83 (0.75, 0.89)	0.05	0.14
WEMT (ms)	0.04	0.75	0.75 (0.64, 0.83)	0.76	2.11
A1DLAr (mm^2^)	0.13	0.66	0.65 (0.49, 0.77)	0.02	0.06
HCDLAr (mm^2^)	0.14	0.92	0.92 (0.88, 0.95)	0.23	0.64
A2DLAr (mm^2^)	0.18	0.70	0.70 (0.56, 0.80)	0.04	0.11
A1dArcL (mm)	0.00	0.65	0.65 (0.49, 0.77)	0.00	0.00
HCdArcL (mm)	−0.15	0.85	0.85 (0.78, 0.90)	0.01	0.03
A2dArcL (mm)	0.00	0.65	0.65 (0.49, 0.77)	0.00	0.00
dArcLM (mm)	−0.2	0.90	0.89(0.85, 0.93)	0.01	0.03
New Variables					
Max Inverse Radius (mm^−1^)	0.11	0.86	0.85 (0.78, 0.90)	0.01	0.03
DA Ratio Max [2 mm]	0.08	0.91	0.91 (0.87, 0.94)	0.18	0.50
Pachy Slope (µm)	0.19	0.96	0.96 (0.95, 0.98)	2.50	6.93
DA Ratio Max [1 mm]	0.03	0.91	0.91 (0.87, 0.94)	0.03	0.08
ARTh	0.17	0.90	0.90 (0.85, 0.93)	45.08	124.87
bIOP (mmHg)	0.10	0.93	0.93 (0.90, 0.96)	0.71	1.97
Integrated Radius (mm^−1^)	0.10	0.92	0.92 (0.88, 0.94)	0.39	1.08
SP A1	0.12	0.92	0.92 (0.88, 0.95)	6.07	16.81
CBI	1.80	0.81	0.81 (0.72, 0.87)	0.06	0.17

CV: coefficient of variation, ICC: interclass correlation coefficient, CI: Confidence interval, RC: repeatability coefficient, S_w_: within-subject SD.

**Table 3:** Repeatability of the Corvis ST parameters in keratoconus eyes.

Parameters	CV (%)	Cronbach’ α	ICC (95% *CI*)	S_w_	RC
Established Variables					
IOP (mmHg)	0.19	0.95	0.95 (0.92, 0.97)	1.00	2.77
CCT (µm)	0.09	0.99	0.99 (0.99, 1.00)	5.93	16.43
DA Max (mm)	0.11	0.94	0.94 (0.91, 0.96)	0.05	0.14
A1T (ms)	0.04	0.94	0.94 (0.92, 0.96)	0.11	0.30
A1V (m/s)	0.18	0.93	0.93 (0.89, 0.95)	0.01	0.03
A2T (ms)	0.02	0.91	0.91 (0.87, 0.94)	0.22	0.61
A2V (m/s)	−0.16	0.90	0.91 (0.86, 0.94)	0.03	0.08
HCT (ms)	0.02	0.55	0.54 (0.33, 0.69)	0.45	1.25
PD (mm)	0.05	0.92	0.92(0.88, 0.95)	0.12	0.33
Radius (mm)	0.18	0.94	0.94 (0.91, 0.96)	0.41	1.14
A1DA (mm)	0.07	0.73	0.73 (0.61, 0.82)	0.01	0.03
HCDA (mm)	0.11	0.94	0.94 (0.91, 0.96)	0.05	0.14
A2DA (mm)	0.18	0.87	0.87 (0.81, 0.91)	0.05	0.14
A1DLL (mm)	0.13	0.67	0.66 (0.50, 0.78)	0.27	0.75
HCDLL (mm)	0.22	0.67	0.67 (0.50, 0.79)	1.06	2.94
A2DLL (mm)	0.29	0.54	0.53 (0.27, 0.70)	1.06	2.94
A1DLA(mm)	0.09	0.93	0.93 (0.89, 0.95)	0.01	0.03
HCDLA (mm)	0.13	0.97	0.96 (0.95, 0.98)	0.04	0.11
A2DLA (mm)	0.17	0.64	0.64 (0.47, 0.76)	0.02	0.06
DLAML (mm)	0.12	0.95	0.95 (0.93, 0.97)	0.05	0.14
DLAMT (ms)	0.03	0.30	0.31 (−0.01, 0.54)	0.70	1.94
WEMA (mm)	0.25	0.88	0.88(0.82, 0.92)	0.04	0.11
WEMT (ms)	0.03	0.62	0.60 (0.42, 0.73)	0.85	2.35
A1DLAr (mm^2^)	0.16	0.82	0.82 (0.73, 0.88)	0.02	0.06
HCDLAr (mm^2^)	0.15	0.94	0.93 (0.90, 0.96)	0.25	0.69
A2DLAr (mm^2^)	0.28	0.53	0.53 (0.32, 0.69)	0.08	0.22
A1dArcL (mm)	0.00	0.81	0.82 (0.73, 0.88)	0.00	0.00
HCdArcL (mm)	−0.36	0.48	0.47 (0.23, 0.65)	0.05	0.14
A2dArcL (mm)	−0.50	0.82	0.82 (0.74, 0.88)	0.01	0.03
dArcLM (mm)	−0.21	0.78	0.78 (0.68, 0.85)	0.03	0.08
New Variables					
Max Inverse Radius (mm^−1^)	0.17	0.79	0.79 (0.69, 0.86)	0.03	0.08
DA Ratio Max [2 mm]	0.24	0.98	0.98 (0.96, 0.98)	0.4	1.11
Pachy Slope (µm)	0.45	0.98	0.98 (0.97, 0.99)	8.62	23.88
DA Ratio Max[1 mm]	0.06	0.96	0.96 (0.94, 0.97)	0.04	0.11
ARTh	0.53	0.99	0.99 (0.98, 0.99)	24.58	68.09
bIOP (mmHg)	0.16	0.94	0.94 (0.92, 0.96)	0.99	2.74
Integrated Radius (mm^−1^)	0.22	0.98	0.98 (0.97, 0.98)	0.72	1.99
SP A1	0.32	0.98	0.98 (0.97, 0.99)	5.56	15.40
CBI	0.34	0.95	0.95 (0.93, 0.97)	0.11	0.30

CV: coefficient of variation, ICC: interclass correlation coefficient, CI: Confidence interval, RC: repeatability coefficient, S_w_: within-subject SD

### Relationship between baseline factors and new Corvis ST parameters repeatability

The correlations between the baseline factors (age, AL, spherical equivalent, astigmatism, gender, Kmean, IOP and CCT) and within-subject SDs of three repeated measurements were analyzed, and the detail results of normal eyes and keratoconus eyes were shown in Supplementary Tables 2 and 3, respectively. The beta coefficients of baseline factors for new parameters repeatability were shown in Table 4. In normal eyes, the ICC of Max Inverse Radius was 0.85, and was associated with spherical equivalent (β = 0.001). The ICC of DA Ratio Max [2 mm] was 0.91, and was associated with AL (β = −0.027), spherical equivalent (β = 0.015) and CCT (β = −0.001). The ICC of Pachy Slope was 0.96, and was associated with AL (β = −0.292) and spherical equivalent (β = 0.177). The ICC of DA Ratio Max [1 mm] was 0.91, and was associated with AL (β = −0.006), spherical equivalent (β = 0.003) and Kmean (β = 0.003). The ICC of ARTh was 0.90, and was associated with gender (β = −0.023) and IOP (β = 4.516). The ICC of bIOP was 0.93, and was associated with AL (β = −0.119) and spherical equivalent (β = 0.064). The ICC of Integrated Radius was 0.92, and was associated with spherical equivalent (β = 0.033). The ICC of SP A1 was 0.92, and was not associated with the baseline factors (*P* > 0.05). The ICC of CBI was 0.81, and was associated with AL (β = −0.016), spherical equivalent (β = 0.008), astigmatism (β = −0.021), Kmean (β = 0.011), IOP (β = −0.010) and CCT (β = −0.001).

**Table 4:** Relationship between baseline factors and within-subject SDs of new Corvis ST paramaters.

Parameters	Age	AL	Spherical equivalent	Astigmatism	Gender	Kmean	IOP	CCT
**Normal eyes**								
Max Inverse Radius (mm^−1^)	0.000	−0.001	**0.001** ^*^	0.002	0.001	0.000	0.000	0.000
DA Ratio Max [2 mm]	−0.004	**−0.027** ^*^	**0.015** ^*^	0.029	0.002	0.018	0.000	**−0.001** ^*^
Pachy Slope (µm)	−0.018	**−0.292** ^*^	**0.177**	−0.481	0.006	0.065	−0.030	0.002
DA Ratio Max [1 mm]	0.000	**−0.006** ^*^	**0.003** ^*^	0.000	0.000	**0.003** ^*^	−0.001	0.000
ARTh	−0.216	2.174	−1.832	−8.531	**−0.023** ^*^	0.151	**4.516** ^*^	0.064
bIOP (mmHg)	0.001	**−0.119** ^*^	**0.064** ^*^	0.082	−0.057	0.073	−0.025	−0.002
Integrated Radius (mm^−1^)	−0.013	−0.044	**0.033** ^*^	0.057	−0.060	0.015	−0.008	0.000
SP A1	0.086	−0.445	0.186	0.747	0.848	0.306	−0.106	0.021
CBI	0.001	**−0.016** ^*^	**0.008** ^*^	**−0.021** ^*^	−0.002	**0.011** ^*^	**−0.010** ^*^	**−0.001** ^*^
**Keratoconus eyes**								
Max Inverse Radius (mm^−1^)	0.000	0.002	−0.001	0.000	−0.006	0.000	0.001	0.000
DA Ratio Max[2 mm]	−0.010	−0.012	−0.002	0.006	0.028	**0.017** ^*^	−0.018	**−0.002** ^*^
Pachy Slope (µm)	**−0.247** ^*^	**−0.824** ^*^	−0.221	−0.457	0.030	**0.453** ^*^	**−0.749** ^*^	**−0.048** ^*^
DA Ratio Max[1 mm]	0.000	0.000	0.000	0.000	−0.006	0.001	−0.001	0.000
ARTh	0.320	1.243	0.665	1.159	0.558	−0.551	**1.773** ^*^	**0.167** ^*^
bIOP (mmHg)	0.001	−0.049	0.008	−0.013	0.185	0.010	0.051	0.001
Integrated Radius (mm^−1^)	−0.020	−0.028	−0.007	−0.013	0.045	**0.028**	**−0.053**	**−0.004**
SP A1	0.011	−0.207	**0.185** ^*^	0.146	0.564	0.037	0.176	**0.016**
CBI	0.002	0.005	0.001	0.001	0.000	−0.002	**0.013**	**0.001**

AL: axial measurement, Kmean: mean keratometry, IOP: intraocular pressure, CCT: central corneal thickness

*Means *P < *0.05.

In keratoconus eyes, the ICC of Max Inverse Radius was 0.79, and was not associated with the baseline factors (*P* > 0.05). The ICC of DA Ratio Max [2 mm] was 0.98, and was associated with Kmean (β = 0.017) and CCT (β = −0.002). The ICC of Pachy Slope was 0.98, and was associated with age (β = −0.247), AL (β = −0.824), Kmean (β = 0.453), IOP (β = −0.749) and CCT (β = −0.048). The ICC of DA Ratio Max [1 mm] was 0.96, and was not associated with the baseline factors (*P* > 0.05). The ICC of ARTh was 0.99, and was associated with IOP (β = 1.773) and CCT (β = 0.167). The ICC of bIOP was 0.94, and was not associated with the baseline factors (*P* > 0.05). The ICC of Integrated Radius was 0.98, and was associated with Kmean (β = 0.028), IOP (β = −0.053) and CCT (β = −0.004). The ICC of SP A1 was 0.98, and was associated with spherical equivalent (β = 0.185) and CCT (β = 0.016). The ICC of CBI was 0.95, and was associated with IOP (β = 0.013) and CCT (β = 0.001).

### ROC curve analyses of new Corvis ST parameters

**Figure 3:**
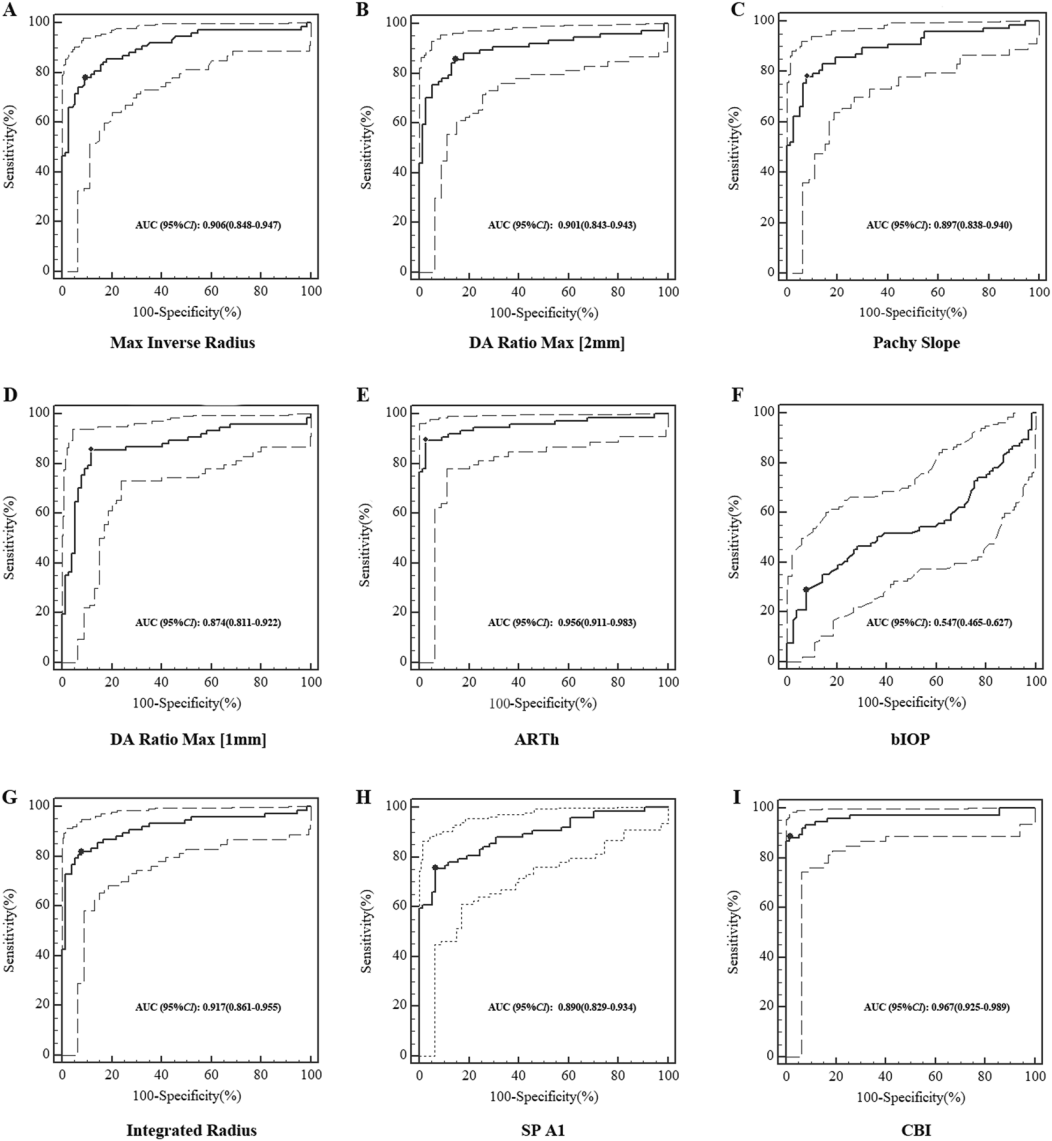
Diagnostic values of new Corvis ST parameters. The ROC analysis for detection of keratoconus using Max Inverse Radius (**A**), DA Ratio Max [2 mm] (**B**), Pachy Slope (**C**), DA Ratio Max [1 mm] (**D**), ARTh (**E**), bIOP (**F**), Integrated Radius (**G**), SP A1 (**H**) and CBI (**I**).

Supplementary Table 4 indicated the differences of Corvis ST parameters between normal eyes and keratoconus eyes. The new parameters except for bIOP were different when adjusting for age, CCT and IOP (Table 5). Compared to normal eyes, the keratoconus eyes had lower values of ARTh, SP A1 and higher values of Max Inverse Radius, DA Ratio Max[2 mm], Pachy Slope, DA Ratio Max[1 mm], Integrated Radius and CBI (All *P* < 0.05). Further ROC curve analyses of Corvis ST parameters were shown in Supplementary Table 5. Both ARTh and CBI had high Youden index (0.870). With a CBI cut-off value of 0.44, 87.0% of the keratoconus were correctly classified with 88.31% sensitivity and 98.70% specificity. With the ARTh cut-off value of 379.29, 87.0% of the keratoconus were correctly classified with 89.61% sensitivity and 97.40% specificity (*P* < 0.001, Fig. 3).

**Table 5:** Comparison of new Corvis ST measurements between normal eyes and keratoconus eyes.

Parameters	Normal eyes	Keratoconus eyes	t	*P*	Adjusted P^*^
Max Inverse Radius (mm^−1^)	0.18 ± 0.02	0.24 ± 0.04	−10.600	<0.001	<0.001
DA Ratio Max[2 mm]	4.63 ± 0.38	6.27 ± 1.48	−9.420	<0.001	0.003
Pachy Slope (µm)	42.71 ± 7.98	77.02 ± 34.89	−8.412	<0.001	<0.001
DA Ratio Max [1 mm]	1.62 ± 0.05	1.74 ± 0.11	−9.263	<0.001	0.017
ARTh	495.22 ± 84.02	234.90 ± 124.62	15.199	<0.001	<0.001
bIOP (mmHg)	14.96 ± 1.57	14.64 ± 2.36	0.994	0.322	0.635
Integrated Radius (mm^−1^)	9.01 ± 0.86	12.58 ± 2.78	−10.772	<0.001	<0.001
SP A1	102.32 ± 12.55	69.90 ± 22.44	11.066	<0.001	<0.001
CBI	0.05 ± 0.09	0.85 ± 0.29	−22.803	<0.001	<0.001

*Adjusted for age, CCT and IOP.

## Discussion

The corneal biomechanical properties were important in evaluating the ophthalmic examinations before refractive surgery and detecting keratoconus. The current study found that the repeatability of 39 Corvis ST parameters in normal eyes and keratoconus eyes were acceptable and new Corvis ST measurements could help to diagnose keratoconus eyes in clinical application.

The current study showed that the new Corvis ST measurements had relatively high ICC and low RC values, which was consistent with previous study to some extent. Compared with the study conducted in 61 emmetropia subjects, our study found higher repeatability values of CCT, A1V, A2T, HCT, PD, A2DA, and A1DLL parameters, but lower repeatability values of IOP, DA Max, A1T, A2V, A1DA and A2DLL parameters^24^. Bak-Nielsen *et al*.^13^ studied the considerable variation in repeatability of Corvis indices and found the RC values of IOP, CCT, A1T, A1V, A2T and A2V were 1.2, 14, 0.2, 0.1, 0.5 and 0.1, respectively, which was almost in accordance with current results. In addition, studies performed separately by Chen *et al*.^25^, Noor *et al*.^12^ and Wu *et al*.^26^ found that Corvis ST measurements for IOP, CCT, deformation amplitude indices, PD and other corneal parameters were reliable. The controversial results might be attributed to different software versions and different populations. The repeatability research of the Corvis ST parameters in keratoconus eyes was still limit. Only Ye *et al*.^16^ reported that the ICC values of IOP, CCT, A1DLL, A2DLL, A1V, A2V, PD and Radius were 0.78, 0.98, 0.30, 0.32, 0.81, 0.50, 0.56 and 0.20 in keratoconus eyes, which were lower than those in our study. Considering there were 12 keratoconus patients in Ye *et al*.^16^ study, and the sample size discrepancy might cause this phenomenon. Thus, multi-center researches need to be conducted in further study to certify the repeatability of corneal biomechanics parameters.

Recently, new indices of Corvis ST have been used in clinical examination^18^. However, there was still limited study related to the repeatability of new parameters. The current study found DA Ratio Max [2 mm], Pachy Slope, DA Ratio Max [1 mm], ARTh, bIOP, Integrated Radius, and SP A1 have excellent repeatability both in normal and keratoconus eyes. The results were consistent with Lopes *et al*.^14^ who demonstrated that the new variables presented good precision in healthy eyes, and the corresponding RCs of DA Ratio Max [2 mm], DA Ratio Max [1 mm], integrated radius, Max Inverse Radius, and SP A1 were 0.3601, 0.05263, 0.7202 and 0.00831, respectively. The CBI repeatability in keratoconus eyes was higher than that in normal eyes. The potential reason was that the CBI was calculated by a logistic regression analysis, and the new software could only show a number range of 0–1.

Previous studies found that age, IOP and other basic characters were significantly associated with corneal biomechanical parameters^27,28^. In addition, Miki *et al*.^15^ found that age, AL and CCT had significantly influences on the repeatability of several corneal deformation parameters in healthy subjects. However, the study that explored the effect of baseline factors on the new Corvis ST parameters repeatability in keratoconus eyes has not been reported. The current study indicated that age, AL, spherical equivalent, astigmatism, gender, Kmean, IOP and CCT could affect the repeatability of Corvis ST parameters. The correlations in normal eyes and keratoconus eyes were not absolutely same, and there might be the differences of clinical characters in two groups. Previous studies showed that keratoconus manifested with progressive myopia, serious irregular astigmatism, great values of AL and Kmean, thin cornea thickness, and poor visual acuity^29,30^. Thus, the baseline factors of subjects should be considered when the Corvis ST parameters were used in clinical application.

The Corvis ST Software is a relatively new device that describes the cornea biomechanical characters, and provides new parameters in diagnosing keratoconus^31^. The Max Inverse Radius is defined as the maximum value of radius of curvature during concave phase of the deformation^32^. The DA ratio Max [1 mm] and DA ratio Max [2 mm] are deformation amplitude measured at 1 or 2 mm from the center, and a greater value describes a less resistant for the cornea deformation or a softer cornea. The current study found the values of Max Inverse Radius, DA ratio Max [2 mm], and DA ratio Max [1 mm] in keratoconus eyes were higher than those in normal eyes, which was consistent with Chan *et al*.^17^ finding. In addition, Chan *et al*.^17^ reported DA Ratio Max [2 mm] (AUC = 0.946), DA Ratio Max [1 mm] (AUC = 0.937), and Max Inverse Radius (AUC = 0.954) offered sufficient discriminative ability in diagnosing keratoconus eyes. Our study had a slightly low AUC value, which might be due to the inconsistent criteria of keratoconus. The Pachy Slope reflects the cornea thickness difference from the center to the periphery, and the study related to the parameter has not been reported. We found the keratoconus eyes had higher values than normal eyes, and age was only found negatively associated with Pachy Slope in keratoconus eyes. The potential reason was that keratoconus patients tend to be young, and the corneal stiffness would change with the age increased^33^. The ARTh is calculated by the division between corneal thickness at the thinnest point and pachy metric progression index, and lower value means a faster thickness increase toward the periphery or a thinner cornea^18^. We found keratoconus eyes had lower ARTh value than normal eyes, the Youden index was 0.87 and the AUC was 0.956 at a cut-off value of 379.29. The distinguishing ability of ARTh was effective, which was similar to other studies^34–36^. Hwange *et al*.^34^ reported the AUC of ARTh max (AUC = 0.739) could distinguish normal eyes from minimally affected eyes of patients with highly asymmetric keratoconus. Eman *et al*.^35^ also found ARTh (AUC = 0.88) was a highly sensitive objective parameter in the fellow eye of unilateral keratoconus. However, Shajari *et al*.^36^ demonstrated ARTh max (AUC = 0.613) was no well in differentiating populations at early stages of keratoconus. Considering the discrepancy among different studies, further study of ARTh detection ability would be conducted in the future. The bIOP was derived by finite element simulations that took into account the effects of age, CCT and other dynamic corneal response parameters. The SP A1 is a novel stiffness parameter that defined as resultant pressure divided by deflection at the first applanation, and the force between the external air pressure and IOP were balance at that time^18^. The CBI was developed based on logistic regression analysis considering the deformation response indices and corneal thickness information. The current study showed the diagnostic ability was 0.870 with 98.70% specificity, 88.31% sensitivity and 0.443 CBI cut-off value. Vinciguerra *et al*.^18^ found the keratoconus was corrected with 100% specificity and 94.1% sensitivity when the CBI cut-off value was 0.5. Wang *et al*.^17^ found the AUC of CBI was 0.785 in differentiating keratoconus from normal eyes. The CBI and ARTh parameters have the highest Youden index compared with other parameters, which indicated they have good screening ability of detecting keratoconus eyes from normal eyes. In addition, the Youden indexes of DA Ratio Max [1 mm], Integrated Radius, Radius, DA Ratio Max [2 mm] and Pachy Slope were also above 0.70, and further multi-center studies of their diagnostic abilities are warranted in later researches.

The current study investigated the repeatability of the new Corvis ST parameters in normal and keratoconus eyes. However, it needs to be considered that the current study subjects were mostly from Henan Province of China. Therefore, the results can not directly be extrapolated to other regions and further multicenter study should be conducted in the later study.

In conclusion, this study indicated that the repeatability of Corvis ST parameters were acceptable both in normal and keratoconus eyes, and baseline factors could affect the repeatability of new parameters. Furthermore, the new Corvis ST parameters could effectively differentiate the keratoconus from normal eyes, which might help clinicians to diagnose keratoconus.

## Supplementary information

Supplementary Table

## Data Availability

All relevant data are included in the paper and its Supporting Information files. Contact to Dr. Shengwei Ren (shengweiren1984@163.com) for additional information regarding data access.
