# Supplementary material for: Repeatability and comparison of new Corvis ST parameters in normal and keratoconus eyes
Source: Sci Rep. 2019 Oct 25;9:15379. doi: 10.1038/s41598-019-51502-4 (PMC6814725; doi:10.1038/s41598-019-51502-4)
Supplement: Supplementary file 1 — Supplementary Table [file 41598_2019_51502_MOESM1_ESM.pdf]

Title:

Repeatability and comparison of new Corvis ST parameters in normal and keratoconus eyes

Authors:

Kaili Yang[1], Liyan Xu[1], Qi Fan[1], Dongqing Zhao[1*], Shengwei Ren[1*]

Authors affiliations:

[1] Henan Provincial People’s Hospital, Henan Eye Hospital, Henan Eye Institute, People's Hospital of Zhengzhou University, School of Clinical Medicine, Henan University, Zhengzhou, 450003, China

Supplementary Table 1 The list of abbreviations

Abbreviations	Description
A1DA	deformation amplitude at the first applanation
A1dArcL	delta arc length at the first applanation
A1DLA	deflection amplitude at the first applanation
A1DLAr	deflection area at the first applanation
A1DLL	deflection length at the first applanation
A1T	time from the initiation of air puff until the first applanation
A1V	corneal velocity at the first applanation
A2DA	deformation amplitude at the maximum deformation
A2dArcL	delta arc length at the second applanation
A2DLA	deflection amplitude at the second applanation
A2DLAr	deflection area at the second applanation
A2DLL	deflection length at the second applanation
A2T	time from the initiation of air puff until the second applanation
A2V	corneal velocity at the sccond applanation
AL	axial measurement
ARTh	ambrósio's relational thickness horizontal
AUC	area under the ROC curve
BCVA	best corrected visual acuity
bIOP(mmHg)	biomechanical corrected intraocular pressure
CBI	corvis biomechanical index
CCT	central corneal thickness
Corvis ST	corneal visualisation scheimpflug technology
CV	coefficient of variation
DA Max	max deformation amplitude
DA Ratio Max[1mm]	the ratio between deformation amplitude at the apex 1mm from central cornea
DA Ratio Max[2mm]	the ratio between deformation amplitude at the apex 2mm from central cornea
dArcLM	delta arc length max
DLAML	max length at deflection amplitude
DLAMT	max time at deflection amplitude
HCDA	deformation amplitude at the second applanation
HCdArcL	delta arc length at the maximum deformation
HCDLA	deflection amplitude at the maximum deformation
HCDLAr	deflection area at the maximum deformation
HCDLL	deflection length at the maximum deformation
HCT	time from the initiation of air puff until the maximum deformation
ICC	interclass correlation coefficient
IOP	intraocular pressure
Kf	flat keratometry
Kmean	mean keratometry
Ks	steep keratometry
PD	peak distance
Radius	radius of curvature
RC	repeatability coefficient
ROC	receiver operating characteristic
S_w	within-subject standard deviation
SD	standard deviation
SP A1	stiffness parameter at first applanation
WEMA	max amplitude of whole eye movement
WEMT	max time of whole eye movement

Supplementary Table 2 Relationship between baseline factors and within-subject SDs of Corvis ST paramaters in normal eyes

**Parameters**	**Age**		**AL**		**Spherical equivalent**		**Astigmatism**		**Gender**		**Kmean**		**IOP**		**CCT**	
	**β**	***P***	**β**	***P***	**β**	***P***	**β**	***P***	**β**	***P***	**β**	***P***	**β**	***P***	**β**	***P***
**Established Variables**																
IOP(mmHg)	0.006	0.731	0.044	0.492	0.027	0.452	0.156	0.271	-0.206	0.127	-0.082	0.125	-0.004	0.914	-0.002	0.439
CCT(μm)	-0.045	0.582	0.007	0.981	-0.114	0.472	-0.123	0.847	0.247	0.688	0.017	0.945	-0.036	0.847	-0.014	0.134
DA Max(mm)	0.000	0.985	**-0.007**	**0.010**	**0.003**	**0.027**	0.003	0.616	0.003	0.607	0.002	0.291	**-0.003**	**0.050**	0.000	0.151
A1T(ms)	-0.001	0.569	**-0.020**	**<0.001**	**0.010**	**0.002**	0.003	0.795	-0.007	0.551	**0.011**	**0.022**	-0.003	0.479	0.000	0.476
A1V(m/s)	0.000	0.944	**-0.001**	**0.041**	0.000	0.083	0.001	0.549	0.000	0.653	**0.001**	**0.036**	0.000	0.676	0.000	0.316
A2T(ms)	-0.003	0.308	**-0.030**	**0.005**	**0.016**	**0.006**	0.023	0.341	0.001	0.975	0.011	0.234	-0.010	0.161	0.000	0.251
A2V(m/s)	0.000	0.185	0.000	0.917	0.000	0.579	0.005	0.073	-0.004	0.084	0.000	0.693	0.000	0.758	0.000	0.392
HCT(ms)	-0.007	0.298	-0.011	0.646	0.013	0.278	**0.112**	**0.024**	-0.041	0.385	0.001	0.949	0.000	0.991	0.001	0.210
PD(mm)	-0.002	0.265	-0.016	0.025	**0.011**	**0.008**	0.012	0.489	0.000	0.979	0.005	0.467	-0.007	0.15	0.000	0.086
Radius(mm)	-0.012	0.106	-0.037	0.161	0.022	0.134	0.078	0.178	0.036	0.515	0.002	0.910	0.000	0.986	0.001	0.522
A1DA(mm)	0.000	0.367	-0.001	0.192	0.000	0.385	0.001	0.548	0.001	0.510	0.000	0.310	0.000	0.553	0.000	0.942
HCDA(mm)	0.000	0.985	**-0.007**	**0.010**	**0.003**	**0.027**	0.003	0.616	0.003	0.607	0.002	0.291	**-0.003**	**0.050**	0.000	0.155
A2DA(mm)	0.000	0.982	-0.002	0.426	0.001	0.349	0.004	0.460	-0.005	0.414	0.003	0.215	-0.001	0.465	0.000	0.734
A1DLL(mm)	-0.003	0.527	-0.002	0.935	-0.004	0.700	-0.071	0.075	0.000	0.990	0.001	0.967	-0.014	0.213	-0.001	0.154
HCDLL(mm)	0.004	0.751	-0.064	0.173	-0.016	0.555	-0.092	0.395	0.004	0.968	0.025	0.547	-0.052	0.098	**-0.004**	**0.011**
A2DLL(mm)	0.010	0.629	-0.065	0.385	-0.043	0.303	-0.183	0.277	-0.015	0.929	0.082	0.200	-0.052	0.287	-0.002	0.556
A1DLA(mm)	0.000	0.707	0.000	0.461	0.000	0.802	0.000	0.731	0.001	0.291	0.000	0.299	0.000	0.903	0.000	0.639
HCDLA(mm)	0.000	0.936	-0.005	0.058	**0.003**	**0.032**	0.002	0.703	-0.002	0.693	0.002	0.390	**-0.003**	**0.037**	0.000	0.180
A2DLA(mm)	0.000	0.324	0.000	0.697	0.000	0.718	0.001	0.441	**-0.003**	**0.045**	0.000	0.996	0.000	0.238	0.000	0.502
DLAML(mm)	0.000	0.682	**-0.004**	**0.036**	0.002	0.083	-0.002	0.744	-0.003	0.565	0.001	0.540	**-0.003**	**0.044**	0.000	0.534
DLAMT(ms)	-0.001	0.940	-0.029	0.342	0.008	0.647	**0.184**	**0.009**	-0.029	0.676	0.016	0.544	-0.018	0.395	**0.002**	**0.044**
WEMA(mm)	0.000	0.620	-0.002	0.424	0.002	0.286	0.006	0.347	-0.005	0.414	0.003	0.195	-0.001	0.771	0.000	0.760
WEMT(ms)	0.006	0.731	0.044	0.492	0.027	0.452	0.156	0.271	-0.206	0.127	-0.082	0.125	-0.004	0.914	-0.002	0.439
A1DLAr(mm^2)	0.000	0.731	0.000	0.570	0.000	0.637	0.001	0.665	-0.001	0.611	-0.001	0.436	0.000	0.866	0.000	0.317
HCDLAr(mm^2)	-0.001	0.805	-0.020	0.143	0.014	0.076	0.002	0.96	-0.023	0.447	0.009	0.477	-0.016	0.083	0.001	0.233
A2DLAr(mm^2)	0.000	0.540	0.001	0.621	0.000	0.751	0.006	0.313	-0.010	0.081	-0.001	0.800	-0.002	0.331	**0.000**	**0.028**
A1dArcL(mm)	0.000	0.902	0.000	0.151	0.000	0.244	0.000	0.739	0.000	0.947	0.000	0.282	0.000	0.119	**0.000**	**0.029**
HCdArcL(mm)	0.000	0.915	-0.001	0.104	0.001	0.052	0.000	0.836	0.001	0.769	0.000	0.690	**-0.001**	**0.121**	0.000	0.411
A2dArcL(mm)	0.000	0.069	0.000	0.695	0.000	0.773	0.000	0.587	0.000	0.754	0.000	0.519	0.000	0.341	0.000	0.128
dArcLM(mm)	0.000	0.954	-0.001	0.327	0.000	0.842	0.001	0.531	0.000	1.000	0.001	0.052	-0.001	0.181	0.000	0.271
**New Variables**																
Max Inverse Radius(mm^{-1})	0.000	0.242	-0.001	0.195	**0.001**	**0.041**	0.002	0.212	0.001	0.614	0.000	0.681	0.000	0.775	0.000	0.987
DA Ratio Max[2mm]	-0.004	0.214	**-0.027**	**0.021**	**0.015**	**0.023**	0.029	0.287	0.002	0.928	0.018	0.074	0.000	0.977	**-0.001**	**0.042**
Pachy Slope(μm)	-0.018	0.617	**-0.292**	**0.017**	**0.177**	**0.012**	-0.481	0.094	0.006	0.983	0.065	0.551	-0.030	0.719	0.002	0.587
DA Ratio Max[1mm]	0.000	0.604	**-0.006**	**0.001**	**0.003**	**0.002**	0.000	0.970	0.000	0.937	**0.003**	**0.034**	-0.001	0.278	0.000	0.296
ARTh	-0.216	0.776	2.174	0.414	-1.832	0.214	-8.531	0.150	**-11.923**	**0.034**	0.151	0.947	**4.516**	**0.008**	0.064	0.473
bIOP(mmHg)	0.001	0.915	**-0.119**	**0.006**	**0.064**	**0.009**	0.082	0.416	-0.057	0.559	0.073	0.057	-0.025	0.398	-0.002	0.124
Integrated Radius(mm^{-1})	-0.013	0.085	-0.044	0.076	**0.033**	**0.022**	0.057	0.330	-0.060	0.279	0.015	0.501	-0.008	0.656	0.000	0.971
SP A1	0.086	0.378	-0.445	0.178	0.186	0.323	0.747	0.325	0.848	0.245	0.306	0.284	-0.106	0.635	0.021	0.068
CBI	0.001	0.430	**-0.016**	**0.003**	**0.008**	**0.014**	**-0.021**	**0.027**	-0.002	0.872	**0.011**	**0.034**	**-0.010**	**0.009**	**-0.001**	**<0.001**

AL: axial measurement, Kmean: mean keratometric, IOP: intraocular pressure, CCT: central corneal thickness.

Supplementary Table 3 Relationship between baseline factors and within-subject SDs of Corvis ST paramaters in keratoconus eyes

**Parameters**	**Age**		**AL**		**Spherical equivalent**		**Astigmatism**		**Gender**		**Kmean**		**IOP**		**CCT**	
	**β**	***P***	**β**	***P***	**β**	***P***	**β**	***P***	**β**	***P***	**β**	***P***	**β**	***P***	**β**	***P***
**Established Variables**																
IOP(mmHg)	-0.001	0.961	-0.024	0.550	-0.016	0.306	-0.051	0.052	-0.007	0.963	0.017	0.162	**-0.072**	**0.010**	-0.003	0.075
CCT(μm)	-0.058	0.514	0.008	0.943	-0.169	0.142	**-0.355**	**0.002**	-0.783	0.395	**0.428**	**<0.001**	**-0.244**	**0.032**	-0.015	0.149
DA Max(mm)	0.000	0.888	-0.001	0.651	0.000	0.512	**0.002**	**0.245**	0.009	0.236	**0.001**	**0.151**	**0.000**	**0.758**	0.000	0.717
A1T(ms)	0.001	0.586	-0.005	0.266	0.002	0.237	0.000	0.936	0.021	0.234	0.001	0.671	**0.010**	**0.003**	0.000	0.342
A1V(m/s)	0.000	0.083	0.000	0.463	0.000	0.325	0.000	0.127	0.002	0.355	**0.000**	**0.050**	0.000	0.564	**0.000**	**0.004**
A2T(ms)	-0.004	0.249	-0.001	0.893	0.004	0.278	0.000	0.974	0.050	0.141	0.001	0.801	0.013	0.053	0.000	0.393
A2V(m/s)	0.000	0.364	0.000	0.813	0.000	0.212	-0.001	0.125	0.002	0.502	0.001	0.059	0.000	0.900	0.000	0.655
HCT(ms)	-0.009	0.119	-0.014	0.407	0.001	0.924	-0.006	0.614	-0.047	0.440	0.002	0.691	-0.002	0.842	**0.001**	0.171
PD(mm)	0.000	0.902	-0.001	0.882	0.000	0.992	-0.001	0.732	0.006	0.688	0.000	0.993	**0.006**	**0.033**	0.000	0.057
Radius(mm)	-0.006	0.350	-0.003	0.863	-0.008	0.211	0.003	0.784	-0.007	0.910	0.002	0.750	-0.015	0.205	0.000	0.481
A1DA(mm)	0.000	0.416	0.000	0.400	0.000	0.732	0.000	0.623	0.000	0.913	0.000	0.912	0.000	0.904	0.000	0.879
HCDA(mm)	0.000	0.888	-0.001	0.651	0.000	0.512	0.002	0.245	0.009	0.236	0.001	0.151	0.000	0.758	0.000	0.717
A2DA(mm)	-0.001	0.230	-0.002	0.351	0.000	0.705	0.000	0.849	0.008	0.249	0.000	0.815	0.000	0.917	0.000	0.586
A1DLL(mm)	-0.005	0.233	-0.010	0.418	0.003	0.474	0.006	0.449	-0.026	0.567	0.000	0.922	-0.005	0.583	0.000	0.795
HCDLL(mm)	-0.025	0.145	-0.017	0.706	**-0.038**	**0.042**	0.013	0.685	0.160	0.376	0.013	0.350	-0.064	0.072	**-0.004**	**0.035**
A2DLL(mm)	-0.016	0.292	0.035	0.392	**-0.043**	**0.005**	0.011	0.704	0.215	0.160	0.015	0.220	-0.057	0.057	**-0.003**	**0.044**
A1DLA(mm)	**0.000**	**0.029**	0.000	0.774	0.000	0.763	0.000	0.993	0.000	0.828	0.000	0.091	0.000	0.717	**0.000**	**0.003**
HCDLA(mm)	0.000	0.532	-0.002	0.160	0.001	0.326	0.001	0.133	0.003	0.591	**0.001**	**0.015**	0.002	0.125	0.000	0.607
A2DLA(mm)	-0.001	0.146	0.000	0.941	0.000	0.680	0.000	0.767	0.005	0.287	0.000	0.818	0.001	0.355	0.000	0.529
DLAML(mm)	0.000	0.783	0.000	0.855	0.000	0.887	0.002	0.064	-0.002	0.782	0.001	0.261	0.001	0.359	0.000	0.966
DLAMT(ms)	0.001	0.897	0.020	0.407	-0.015	0.107	0.014	0.391	-0.039	0.667	0.005	0.487	0.009	0.618	0.000	0.712
WEMA(mm)	0.000	0.836	-0.002	0.191	0.000	0.776	0.000	0.984	0.002	0.743	0.000	0.895	-0.002	0.160	0.000	0.721
WEMT(ms)	-0.001	0.961	-0.024	0.550	-0.016	0.306	-0.051	0.052	-0.007	0.963	0.017	0.162	**-0.072**	**0.010**	-0.003	0.075
A1DLAr(mm2)	0.000	0.145	-0.001	0.060	0.000	0.949	**-0.001**	**0.037**	-0.001	0.811	**0.000**	0.229	0.000	0.482	**0.000**	**0.020**
HCDLAr(mm2)	0.001	0.779	0.001	0.897	0.001	0.879	0.005	0.398	-0.011	0.744	0.000	0.885	0.008	0.224	0.001	0.094
A2DLAr(mm2)	-0.003	0.080	0.000	0.936	-0.001	0.614	-0.002	0.527	0.021	0.209	0.001	0.654	0.001	0.693	0.000	0.830
A1dArcL(mm)	**0.000**	**0.021**	0.000	0.306	0.000	0.416	0.000	0.334	0.000	0.908	0.000	0.153	0.000	0.098	**0.000**	**0.032**
HCdArcL(mm)	0.000	0.778	0.001	0.734	-0.001	0.379	0.002	0.461	-0.007	0.550	0.000	0.705	-0.001	0.739	0.000	0.519
A2dArcL(mm)	**0.000**	**0.044**	0.000	0.823	0.000	0.681	0.000	0.183	**0.003**	**0.023**	0.000	0.064	0.000	0.312	0.000	0.170
dArcLM(mm)	0.000	0.719	-0.001	0.417	0.000	0.901	0.000	0.838	-0.004	0.465	0.000	0.646	-0.001	0.403	0.000	0.302
**New Variables**																
Max Inverse Radius(mm^{-1})	0.000	0.757	0.002	0.404	-0.001	0.418	0.000	0.825	-0.006	0.448	0.000	0.771	0.001	0.711	0.000	0.748
DA Ratio Max[2mm]	-0.010	0.076	-0.012	0.449	-0.002	0.773	0.006	0.615	0.028	0.648	**0.017**	**0.001**	-0.018	0.132	**-0.002**	**0.003**
Pachy Slope(μm)	**-0.247**	0.082	**-0.824**	**0.038**	-0.221	0.154	-0.457	0.090	0.030	0.984	**0.453**	**<0.001**	**-0.749**	**0.009**	**-0.048**	**0.003**
DA Ratio Max[1mm]	0.000	0.733	0.000	0.794	0.000	0.783	0.000	0.883	-0.006	0.350	0.001	0.084	-0.001	0.601	0.000	0.222
ARTh	0.320	0.434	1.243	0.280	0.665	0.134	1.159	0.134	0.558	0.896	-0.551	0.110	**1.773**	**0.032**	**0.167**	**<0.001**
bIOP(mmHg)	0.001	0.942	-0.049	0.238	0.008	0.638	-0.013	0.635	0.185	0.224	0.010	0.408	0.051	0.090	0.001	0.433
Integrated Radius(mm^{-1})	-0.020	0.051	-0.028	0.337	-0.007	0.551	-0.013	0.487	0.045	0.673	**0.028**	**0.001**	**-0.053**	**0.010**	**-0.004**	**0.001**
SP A1	0.011	0.876	-0.207	0.289	**0.185**	**0.013**	0.146	0.268	0.564	0.435	0.037	0.533	0.176	0.216	**0.016**	**0.047**
CBI	0.002	0.349	0.005	**0.372**	0.001	0.566	0.001	0.833	0.000	0.985	-0.002	0.253	**0.013**	**0.005**	**0.001**	**0.001**

AL: axial measurement, Kmean: mean keratometric, IOP: intraocular pressure, CCT: central corneal thickness

Supplementary Table 4 Comparison of the Corvis ST measurements between normal eyes and keratoconus eyes

**Parameters**	**Normal eyes**	**Keratoconus eyes**	**t**	***P***	**Adjusted *P****
IOP(mmHg)	14.90±1.62	13.26±2.53	4.812	<0.001	0.207
CCT(μm)	542.76±31.49	485.77±45.09	9.092	<0.001	<0.001
DAMax(mm)	1.12±0.09	1.22±0.13	-5.431	<0.001	0.036
A1T(ms)	7.18±0.20	6.98±0.27	5.261	<0.001	0.046
A1V(m/s)	0.15±0.01	0.17±0.03	-4.987	<0.001	0.319
A2T(ms)	22.23±0.33	22.40±0.43	-2.805	0.006	0.131
A2V(m/s)	-0.29±0.03	-0.31±0.05	3.561	<0.001	0.953
HCT(ms)	17.38±0.41	17.39±0.38	-0.227	0.821	<0.001
PD(mm)	5.11±0.23	5.04±0.24	1.894	0.060	0.001
Radius(mm)	6.82±0.60	5.28±0.97	11.877	<0.001	<0.001
A1DA(mm)	0.13±0.01	0.14±0.01	-6.810	<0.001	0.001
HCDA(mm)	1.12±0.09	1.22±0.13	-5.431	<0.001	0.036
A2DA(mm)	0.40±0.07	0.40±0.07	-0.057	0.955	0.110
A1DLL(mm)	2.27±0.23	2.33±0.30	-1.443	0.151	0.002
HCDLL(mm)	6.36±0.63	5.25±1.17	7.283	<0.001	0.001
A2DLL(mm)	3.41±0.96	3.41±1.00	-0.006	0.995	0.956
A1DLA(mm)	0.09±0.01	0.11±0.01	-8.718	<0.001	<0.001
HCDLA(mm)	0.93±0.09	1.03±0.13	-5.427	<0.001	0.690
A2DLA(mm)	0.11±0.01	0.12±0.02	-4.462	<0.001	0.012
DLAML(mm)	0.95±0.09	1.05±0.13	-5.559	<0.001	0.158
DLAMT(ms)	16.43±0.41	16.38±0.48	0.681	0.497	0.853
WEMA(mm)	0.30±0.06	0.28±0.07	1.032	0.304	0.376
WEMT(ms)	22.37±0.88	22.4±0.77	-0.266	0.791	0.484
A1DLAr(mm^2)	0.16±0.02	0.19±0.03	-7.996	<0.001	<0.001
HCDLAr(mm^2)	3.44±0.47	3.59±0.55	-1.848	0.067	0.072
A2DLAr(mm^2)	0.22±0.04	0.25±0.07	-3.042	0.003	0.066
A1dArcL(mm)	-0.01±0.00	-0.02±0.00	5.805	<0.001	0.001
HCdArcL(mm)	-0.13±0.02	-0.11±0.04	-4.411	<0.001	0.111
A2dArcL(mm)	-0.02±0.00	-0.02±0.01	3.162	0.002	0.034
dArcLM(mm)	-0.15±0.03	-0.14±0.03	-3.490	0.001	0.751

* Adjusted for age, CCT and IOP (except where it is the variable of interest).

Supplementary Table 5 The ROC analysis of Corvis ST parameters in distinguishing keratoconus from normal eyes

**Parameters**	**Cut-off**	**Sensitivity (%)**	**Specificity (%)**	**Youden index**	**AUC (95%*CI*)**
**Established Variables**					
IOP(mmHg)	12.667	44.16	94.81	0.390	0.705(0.626-0.776)
CCT(μm)	501.000	63.64	93.51	0.571	0.846(0.779-0.899)
DA Max(mm)	1.224	44.16	89.61	0.338	0.710(0.632-0.781)
A1T(ms)	7.010	55.84	83.12	0.390	0.719(0.641-0.788)
A1V(m/s)	0.163	53.25	88.31	0.416	0.693(0.614-0.765)
A2T(ms)	22.656	32.47	94.81	0.273	0.640(0.559-0.716)
A2V(m/s)	-0.336	37.66	94.81	0.325	0.640(0.559-0.716)
HCT(ms)	16.786	92.21	12.99	0.052	0.509(0.428-0.591)
PD(mm)	5.018	49.35	68.83	0.182	0.586(0.504-0.665)
Radius(mm)	6.084	80.52	92.21	0.727	0.912(0.855-0.951)
A1DA(mm)	0.131	66.23	85.71	0.520	0.777(0.703-0.840)
HCDA(mm)	1.224	44.16	89.61	0.338	0.710(0.632-0.781)
A2DA(mm)	0.333	25.97	84.42	0.104	0.510(0.428-0.591)
A1DLL(mm)	2.417	45.45	84.42	0.298	0.607(0.525-0.684)
HCDLL(mm)	5.962	66.67	81.82	0.485	0.801(0.729-0.861)
A2DLL(mm)	2.629	74.67	16.88	0.085	0.505(0.422-0..586)
A1DLA(mm)	0.10	67.53	97.40	0.649	0.840(0.773-0.894)
HCDLA(mm)	0.998	59.74	79.22	0.390	0.720(0.642-0.789)
A2DLA(mm)	0.118	41.56	92.21	0.338	0.676(0.596-0.749)
DLAML(mm)	1.007	61.04	79.22	0.403	0.724(0.646-0.792)
DLAMT(ms)	16.311	46.75	63.64	0.104	0.538(0.456-0.619)
WEMA(mm)	0.283	54.55	63.64	0.182	0.563(0.481-0.643)
WEMT(ms)	22.711	40.26	79.22	0.195	0.556(0.473-0.636)
A1DLAr(mm^2)	0.183	53.25	97.40	0.507	0.821(0.751-0.878)
HCDLAr(mm^2)	3.866	32.47	84.42	0.168	0.578(0.495-0.657)
A2DLAr(mm^2)	0.252	37.66	83.12	0.208	0.616(0.535-0.693)
A1dArcL(mm)	-0.017	54.55	93.51	0.481	0.743(0.667-0.810)
HCdArcL(mm)	-0.104	45.45	93.51	0.390	0.722(0.645-0.792)
A2dArcL(mm)	-0.025	40.26	89.61	0.299	0.620(0.538-0.697)
dArcLM(mm)	-0.134	49.35	80.52	0.299	0.675(0.595-0.748)
**New Variables**					
Max Inverse Radius [mm^{-1}]	0.201	77.92	90.91	0.688	0.906(0.848-0.947)
DA Ratio Max [2mm]	4.980	85.71	85.71	0.714	0.901(0.843-0.943)
Pachy Slope (μm)	52.082	77.92	92.21	0.701	0.897(0.838-0.940)
DA Ratio Max [1mm]	1.651	85.71	88.31	0.740	0.874(0.811-0.922)
ARTh	379.294	89.61	97.40	0.870	0.956(0.911-0.983)
bIOP(mmHg)	13.267	28.57	92.21	0.208	0.547(0.465-0.627)
Integrated Radius [mm^{-1}]	9.985	81.82	92.21	0.740	0.917(0.861-0.955)
SP A1	85.297	75.32	93.51	0.688	0.890(0.829-0.934)
CBI	0.443	88.31	98.70	0.870	0.967(0.925-0.989)
